# Auto-immunoproteomics analysis of COVID-19 ICU patients revealed increased levels of autoantibodies related to the male reproductive system

**DOI:** 10.3389/fphys.2023.1203723

**Published:** 2023-07-14

**Authors:** Frank Schmidt, Houari B. Abdesselem, Karsten Suhre, Nishant N. Vaikath, Muhammad U. Sohail, Maryam Al-Nesf, Ilham Bensmail, Fathima Mashod, Hina Sarwath, Joerg Bernhardt, Stephanie Schaefer-Ramadan, Ti-Myen Tan, Priscilla E. Morris, Edward J. Schenck, David Price, Vidya Mohamed-Ali, Mohammed Al-Maadheed, Abdelilah Arredouani, Julie Decock, Jonathan M. Blackburn, Augustine M. K. Choi, Omar M. El-Agnaf

**Affiliations:** ^1^ Proteomics Core, Weill Cornell Medicine—Qatar, Doha, Qatar; ^2^ Proteomics Core Facility, Qatar Biomedical Research Institute (QBRI), Qatar Foundation, Hamad Bin Khalifa University (HBKU), Doha, Qatar; ^3^ Neurological Disorders Research Center, QBRI, HBKU, Qatar Foundation, Doha, Qatar; ^4^ Bioinformatics Core, Weill Cornell Medicine—Qatar, Doha, Qatar; ^5^ Hamad General Hospital, Hamad Medical Corporation, Doha, Qatar; ^6^ Center of Metabolism and Inflammation, Division of Medicine, University College London, London, United Kingdom; ^7^ Institute for Microbiology, University of Greifswald, Greifswald, Germany; ^8^ Department of Genomic Medicine, Weill Cornell Medicine—Qatar, Doha, Qatar; ^9^ Department of Integrative Biomedical Sciences, Faculty of Health Sciences, University of Cape Town, Cape Town, South Africa; ^10^ Sengenics Corporation, Damansara Heights, Kuala Lumpur, Malaysia; ^11^ Institute of Infectious Disease and Molecular Medicine, Faculty of Health Sciences, University of Cape Town, Cape Town, South Africa; ^12^ Department of Medicine, Division of Pulmonary and Critical Care Medicine, New York Presbyterian Hospital—Weill Cornell Medical Center, Weill Cornell Medicine, New York, NY, United States; ^13^ Anti-Doping Laboratory Qatar, Doha, Qatar; ^14^ Diabetes Research Center, Qatar Biomedical Research Institute (QBRI), Hamad Bin Khalifa University (HBKU), Qatar Foundation, Doha, Qatar; ^15^ College of Health and Life Sciences, Hamad Bin Khalifa University (HBKU), Qatar Foundation, Doha, Qatar; ^16^ Translational Cancer and Immunity Center, Qatar Biomedical Research Institute (QBRI), Hamad Bin Khalifa University (HBKU), Qatar Foundation, Doha, Qatar

**Keywords:** COVID-19, autoantibodies, immunoproteomics, SPANXN4, Stk25, male reproductive system

## Abstract

**Background:** Coronavirus disease (COVID-19) manifests many clinical symptoms, including an exacerbated immune response and cytokine storm. Autoantibodies in COVID-19 may have severe prodromal effects that are poorly understood. The interaction between these autoantibodies and self-antigens can result in systemic inflammation and organ dysfunction. However, the role of autoantibodies in COVID-19 complications has yet to be fully understood.

**Methods:** The current investigation screened two independent cohorts of 97 COVID-19 patients [discovery (Disc) cohort from Qatar (case = 49 vs. control = 48) and replication (Rep) cohort from New York (case = 48 vs. control = 28)] utilizing high-throughput KoRectly Expressed (KREX) Immunome protein-array technology. Total IgG autoantibody responses were evaluated against 1,318 correctly folded and full-length human proteins. Samples were randomly applied on the precoated microarray slides for 2 h. Cy3-labeled secondary antibodies were used to detect IgG autoantibody response. Slides were scanned at a fixed gain setting using the Agilent fluorescence microarray scanner, generating a 16-bit TIFF file. Group comparisons were performed using a linear model and Fisher’s exact test. Differentially expressed proteins were used for KEGG and WIKIpathway annotation to determine pathways in which the proteins of interest were significantly over-represented.

**Results and conclusion:** Autoantibody responses to 57 proteins were significantly altered in the COVID-19 Disc cohort compared to healthy controls (*p* ≤ 0.05). The Rep cohort had altered autoantibody responses against 26 proteins compared to non-COVID-19 ICU patients who served as controls. Both cohorts showed substantial similarities (*r* ^2^ = 0.73) and exhibited higher autoantibody responses to numerous transcription factors, immunomodulatory proteins, and human disease markers. Analysis of the combined cohorts revealed elevated autoantibody responses against SPANXN4, STK25, ATF4, PRKD2, and CHMP3 proteins in COVID-19 patients. The sequences for SPANXN4 and STK25 were cross-validated using sequence alignment tools. ELISA and Western blot further verified the autoantigen–autoantibody response of SPANXN4. SPANXN4 is essential for spermiogenesis and male fertility, which may predict a potential role for this protein in COVID-19-associated male reproductive tract complications, and warrants further research.

## Introduction

Coronavirus disease (COVID-19), caused by the novel SARS-CoV-2 virus, has emerged as a global pandemic with severe complications and a high morbidity rate. The disease manifests a wide range of clinical symptoms, which are exacerbated by the overactive and malfunctioning immune system of the host. Despite extensive research on innate and adaptive immune responses in COVID-19, little is known about the role of autoantibodies on disease progression and severe complications.

Infection with SARS-CoV-2 causes various symptoms, with most cases being moderate or asymptomatic and only a smaller proportion advancing to more severe COVID-19 disease (Chen et al., 2020). Many questions about COVID-19 pathophysiology remain open, particularly why some people develop severe disease symptoms while others remain asymptomatic. Furthermore, vaccine efficacy varies between individuals, and only a few therapeutic approaches are investigated. Yuan et al. (2023) reviewed the comprehensive immune response and therapeutic targets in COVID-19, focusing on autoimmune disorders.

Acute respiratory distress syndrome (ARDS) affects a small percentage of patients, whereas others experience persistent lung damage and multi-organ illness that lasts months, even after the virus has been eliminated from the body (Pfortmueller et al., 2020). High expression of angiotensin-converting enzyme 2 (ACE2) receptors in several organs of the body extends the infection beyond the respiratory tract, resulting in complex multi-organ complications (Ashraf et al., 2021). ACE2 receptors are highly expressed in the male reproductive system, and testicular ACE2 and the renin–angiotensin system are implicated in the effect of SARS-CoV-2 on testis function, demonstrating the involvement of SARS-CoV-2 in male fertility, which is one of the unexplained manifestations of COVID-19 (Sun, 2020; Edenfield and Easley, 2022; Ramal-Sanchez et al., 2022). Furthermore, proteomics analysis on the semen of COVID-19 convalescent men has shown disrupted key biological pathways relevant to male reproductive function (Ghosh et al., 2022). Despite extensive research on innate and adaptive immune responses in COVID-19, little is known about the role of autoantibodies in disease progression and severe complications.

Autoantibodies have been identified in a significant proportion of hospitalized COVID-19 patients with a positive correlation with immune responses to SARS-CoV-2 proteins (Chang et al., 2021). Several studies observed a significant increase in a diverse range of autoantibodies against immunomodulatory proteins, α- and ω-interferons, cardiolipin, and prothrombin during antiviral responses in severely ill COVID-19 patients (Bastard et al., 2020; Woodruff et al., 2020a; Woodruff et al., 2020b; Zuo et al., 2020; Garcia-Beltran et al., 2021; Wang et al., 2021). Particularly, autoantibodies against immune-related signaling proteins contributed to COVID-19 pathogenesis by antagonizing the function of the innate immune system (Tay et al., 2020). Although some reports have been found on disease-modifying autoantibody responses, the immunological and clinical consequences of autoantibodies in COVID-19 are yet to be fully understood. Therefore, we screened total IgG autoantibody responses against 1,318 human proteins in COVID-19 patients using KREX Immunome protein-array technology. Sengenics KREX technology employs full-length, naturally folded proteins that allow maximum epitope binding to discover autoantibody biomarker proteins (Blackburn and Hart, 2005). The quantitative signal measured on the arrays for each autoantibody–autoantigen pair is directly proportional to the autoantibody concentration in the blood with higher autoantibody titers to these proteins in COVID-19 patients compared to controls.

Autoantibody-based precision immuno-profiling has previously been shown to aid the discovery of biomarkers of immune-related adverse events, and therapeutic prediction of drug response (Fritzler et al., 2018). In the present study, by utilizing a broad array-based immunoproteomics strategy that simultaneously quantifies autoantibody responses across multiple organ systems in COVID-19 ICU patients and post-recovery cohort, we aimed to better identify novel markers of comorbidities in COVID-19 patients. We identified some novel markers in COVID-19 patients that are also associated with male fertility, such as the sperm protein SPANXN4 (Urizar-Arenaza et al., 2020), the androgenic kinase STK25 (Hammes et al., 2005; Gill-Sharma, 2018), the apoptotic factor ATF4 (Fischer et al., 2004), the calcium channel regulator protein kinase PRKD2 (Dai et al., 2019), and the multi-vesicular protein CHMP3 (Razavi et al., 2017).

## Methods

### Study design, sample collection and processing, and Ethics

We used blood samples and clinical data of patients from two independent COVID-19 cohorts to conduct a comprehensive analysis of autoantibodies using novel KREX technology.

#### Discovery (disc) cohort

The Disc cohort included 49 COVID-19 patients from Qatar admitted to Hamad Medical Corporation hospitals. All recruited patients showed confirmed SARS-CoV-2-positive RT-PCR results of sputum or throat swabs. All patients had severe COVID-19 disease (WHO guidelines) (World Health Organization, 2020) and were admitted to the intensive care unit (ICU). Peripheral blood was collected within 5 to 7 days of admission, processed into plasma and serum, and stored at −80°C, until further analysis. Ethical approval for this cohort was obtained from the Hamad Medical Corporation Institutional Review Board Research Ethics Committee (reference MRC-05-003) and Qatar Biomedical Research Institute—Institutional Review Board (reference QBRI-IRB 2020-06-19).

#### Healthy controls

Age- and gender-matched healthy volunteers (*n* = 48) with no prior COVID-19 infection history and normal oxygen saturation and vital signs were used as controls. The Anti-Doping Laboratory—Qatar recruited them for blood collection. Individuals with medical history or with cognitive disabilities were excluded. All participants (patients and controls) provided written informed consent prior to enrolment in the study.

#### Replication (Rep) cohort

The replication cohort consisted of 48 adult patients admitted to the ICU of New York-Presbyterian Hospital (NYP)/Weill Cornell Medical Center (WCMC) from March to April 2020. All patients were RT-PCR-confirmed SARS-CoV-2-positive and displayed ARDS or pneumonia symptoms. The cohort is part of the Weill Cornell Biobank of Critical Illness, a registry that attempts to recruit and enroll all patients admitted to the WCMC ICU for clinical investigations. The WCMC COVID Institutional Data Repository (COVID-IDR) is a manually abstracted registry of COVID-19 patients developed to record patient demographics and allied health parameters. Laboratory parameters, ventilation records, respiratory variables, and vital signs were recorded and documented at Weill Cornell-Critical carE Database for Advanced Research (WC-CEDAR) (Su et al., 2020). The processes for recruiting patients, collecting data, and processing samples had been previously documented (Finkelsztein et al., 2017; Schenck et al., 2019). Only patients who gave informed consent were included. IRB approvals for this cohort were obtained from NYP/WCMC with reference numbers 20-05022072 and 1405015116.

#### Non-COVID-19 ICU controls

Twenty-eight patients admitted to the NYP Hospital ICU between 2014 and 2019 were included as non-COVID-19 ICU controls for the Rep cohort. These patients were suffering from bacterial sepsis ARDS (*N* = 15), influenza ARDS (*N* = 4), and influenza pneumonia (*N* = 9). Patient recruitment, medical history, and sampling procedures for the non-COVID ICU control cohort are the same as those described for the Rep cohort.

### Sengenics assay description and data pre-processing

The Disc cohort samples were processed at Qatar Biomedical Research Institution (QBRI) for KREX immunoproteomics. The Rep cohort samples were processed at the Sengenics facility in Kuala Lumpur, Malaysia. Samples of the Disc cohort and controls were analyzed for antigen-specific autoantibodies using Immunome protein arrays (Sengenics), developed using KoRectly Expressed (KREX) technology to provide a high-throughput immunoassay based on correctly folded, full-length and functional recombinant human proteins expressed in insect cells, thereby displaying an entire repertoire of continuous and discontinuous epitopes for autoantibody binding (Blackburn et al., 2012; Adeola et al., 2016). The Immunome arrays contain more than 1,600 human antigens, enriched for kinases, signaling molecules, cytokines, interleukins, chemokines, and known autoimmune and cancer antigens. Plasma samples of Rep cohort and non-COVID-19 ICU control patients were processed for autoantibodies on a custom array containing a subset of 1,318 human proteins (Sengenics Immunome protein arrays).

All samples were viral-inactivated in 10% Triton X-100 for 2 h at room temperature. Samples were then diluted in serum albumin buffer (SAB) at optimized dilution (50-fold dilution). Microarray slides were prepared in four-well plates. Samples, including controls, were randomized before being applied to the microarray slides for 2 h. Cy3-labeled IgG antibodies were used as secondary antibodies to detect antibody response. Slides were scanned at a fixed gain setting using the Agilent G4600AD fluorescence microarray scanner, generating a 16-bit TIFF file. A visual quality control check was conducted, and any array showing spot merging or other artifacts was re-assayed. A GAL (GenePix Array List) file containing information regarding the location and identity of all probed spots was used for image analysis. Automatic extraction and quantification of each spot were performed using GenePix Pro 7 software (Molecular Devices), yielding the median foreground and local background pixel intensities for each spot.

Biotinylated human IgG (detected by fluorescently labeled secondary antibody) and biotinylated human anti-IgG (detected only when plasma or serum is added to the slide) were used as positive controls to assess assay integrity. Extrapolated data were then filtered, normalized, and transformed as follows: the median background pixel intensity (RFU) was subtracted from the median foreground pixel intensity (RFU) for each antigen to yield the median net intensity per spot (RFU). CVs were calculated for each antigen based on the quadruplicate technical replica spots on a given array. Antigens with CVs above 20% were flagged, and outlier spots were removed, provided that at least two valid values remained. The net intensity value for each antigen in a given sample was calculated as the mean of the net intensity values for technical replica spots on that array. The data were normalized across replica arrays based on the Cy3–BSA controls, as previously described (Da Gama Duarte et al., 2018). Z-scores were then calculated by subtracting the overall mean antigen intensity (within a single sample) from the net intensity data for each antigen in that sample and dividing that result by the standard deviation of all of the measured net intensities in that sample, according to the formula z = (x–μ)/σ, where x is the net intensity of an antigen in a given sample, m is the mean net intensity calculated across all antigens in that sample, and s is the standard deviation of the net intensities for all antigens in that sample. All downstream statistical analyses were performed based on the calculated z-scores.

### Sequence identity and antigen specificity analysis for selected proteins

We needed to be cautious in directly comparing the results across different antigens on the arrays because the autoantigen–autoantibody response is not always linear and is an indirect way of predicting protein concentrations. A higher autoantibody response may be triggered by several factors, such as increased protein expression or decreased clearance of target proteins and overactivation of the immune system that produces more autoantibodies, since it can be influenced by factors such as B cell activation and sequence identity among proteins that express similar antigen epitopes (Blach-Olszewska and Leszek, 2007). To check this latter possibility, we selected two proteins (SPANXN4 and STK25) that showed the highest autoantibody alterations to perform their sequence alignment and antigen-specificity analysis. The UniProt BLASTP program was used to compare protein sequences. All human and viral protein sequences with more than 50% sequence similarity were aligned for epitope mapping to determine whether the evaluated RFU values were specific to the proteins of interest or could be derived from highly homologous epitopes on other proteins.

### Protein pathway prediction

The assignment of KREX array proteins to functional KEGG categories and their hierarchical organization was displayed using PAVER, a software used for the visualization of Voronoi treemaps (Bernhardt et al., 2009). Any main category is displayed in different colors. The cell sizes were calculated according to the signal intensity of the proteins’ immunofluorescence (highly fluorescent signals give larger cells). Functional enrichment analysis was performed to identify biological functions over-represented in differentially expressed proteins with a *p*-value less than 0.05. Differentially expressed proteins, both upregulated and downregulated, were used separately as proteins of interest, and the proteins detected from all probes were used as the background set. The proteins were further annotated using KEGG and WIKIPathways data before performing Fisher’s exact test to determine pathways in which the proteins of interest were significantly over-represented. This analysis was performed on R 3.6.2 using clusterProfiler 3.14.3. GOSemSim was used to eliminate redundant GO–BP results. Only significantly over-represented pathways with a *p*-value less than 0.05 (−log10 *p*-value cut-off 1.3) are shown.

### Expression and purification of SPANXN4

The BL21 (DE3) competent cell line was transformed with the human SPANXN4 gene in the pExp-GST plasmid. Overnight cultures from a single colony were prepared in LB media (Sigma) with ampicillin and incubated at 37°C. The pre-inoculum was inoculated into 4,000 mL LB media with ampicillin. The culture was grown at 37°C to OD_600_ 0.6–0.8, induced with 0.5 mM IPTG, and incubated at 25°C for ∼20 h. Cells were harvested by centrifugation and resuspended in lysis buffer (phosphate buffer pH 7.4 with 300 mM NaCl, lysozyme 0.1 mg/mL, DNase I 0.01 mg/mL, 0.02 mM MgCl2, and protease inhibitor cocktail 1×). The cells were lysed by sonication (5 s ON and 10 s OFF) for 5 min, 40% amplitude. The lysate was centrifuged at 12,000 rpm for 30 min at 4°C, and the supernatant was purified using an Ni–NTA Sepharose column (GE). The supernatant was passed twice through the column after equilibration with wash buffer (PBS, pH 7.4, containing 10 mM imidazole). The column was washed with 50 column volumes of wash buffer, followed by elution with elution buffer (PBS, pH 7.4 with 500 mM imidazole). The fractions containing SPANXN4 protein were pooled, dialyzed against PBS, and subjected to gel filtration using the HiLoad 26/600 Superdex 200 pg FPLC column. The fractions containing the protein were pooled and quantified using a BCA assay.

### ELISA to measure SAPNXN4 autoantibodies

The 96-well ELISA plate (Nunc, MaxiSorp) was coated with SPANXN4 protein at 1 μg/ml in coating buffer (0.2 M NaHCO_3_, pH 9.6) and incubated at 4°C overnight. The next day, the plate was washed three times with PBST (PBS containing 0.05% Tween-20) and blocked with blocking buffer (2.25% gelatin in PBST) for 1 h at room temperature. After washing, the plate was transferred into a biosafety cabinet for adding serum samples. Serum samples (inactivated with 1% Triton X-100) from COVID-19 patients (*n* = 50)/controls (*n* = 50) were diluted to 1/400 inside the LAF and added to the wells. After adding the serum samples, the plate was incubated at room temperature for 2 h. Following incubation, the plate was washed and incubated with goat anti-human IgG-HRP (1/20,000) antibodies for 1 h at room temperature. Finally, the plate was washed and 100 μL of TMB substrate was added. The plate was incubated for 20 min and the reaction was stopped by adding 50 μL of 0.6 N H_2_SO_4_. The color developed was read at 450 nm using a PerkinElmer plate reader.

### Western blot

SPANXN4 protein (100 ng/well) was loaded onto a 10% NuPAGE Bis-Tris gel (Invitrogen) and ran under reducing conditions. The proteins were transferred onto a nitrocellulose membrane (100 V for 1 h). The membrane was blocked with blocking buffer (5% skimmed milk in PBS-T) for 1 h at room temperature, followed by incubation with 1/200 diluted serum obtained from severe COVID-19 cases (n = 5) or controls (n = 4) for 1 h at room temperature. Following washing with PBS-T, HRP-conjugated goat anti-human IgG (1:20 000 in PBST, Jackson ImmunoResearch Laboratories Inc.) was added and incubated for 1 h at room temperature. After washing again with PBS-T, the membranes were developed with SuperSignal West Pico Substrate (Pierce Biotechnology, Rockford, USA), and the signal was detected using the ChemiDoc gel doc system (BioRad).

### Statistical analysis

Proteins are reported using the symbols of the genes that encode them to offer a precise and uniform nomenclature. Autoantibody response, measured as relative fluorescence units (RFU), was normalized to calculate the z-score. Statistical analysis was performed using R (version 4.1.0) and RStudio (version 1.4.1717). Two kinds of inferential statistical tests were performed to test the hypothesis of whether a given autoantibody was differentially expressed in COVID cases versus controls. First, the means between cases and controls were compared using a linear model, using the z-scored autoantibody responses as dependent variables and the COVID state as an independent variable (coded as 0 = controls and 1 = cases). Notably, this approach is equivalent to conducting an unrelated t-test and that the effect size of the linear model matches the estimated difference of the means in the t-test. Second, binarized autoantibody responses were tested against cases versus controls using Fisher’s exact test. The cutoff for binarization of the autoimmune response was set to 1. As the response is z-scored, all samples with an RFU score above 1 standard deviation from the mean were considered positive for the respective autoantibody, whereas all others were considered negative. The Disc and Rep cohorts were analyzed separately and then merged. Therefore, three sets of *p*-values were obtained for each of the continuous and binarized trait analyses.

The following comparisons were made:

• Differential autoantibody response analysis: COVID-19 cases versus controls for
• Forty-nine Disc COVID-19 patients vs. forty-eight controls.
• Forty-eight Rep COVID-19 patients vs. twenty-eight non-COVID-19 ICU controls.
• A combination of ninety-seven COVID-19 patients vs. seventy-six controls from both Disc and Rep cohorts.
• Pearson’s correlation analysis of 15 Disc cohort patients sampled at the time of ICU admission (T1) and 6 weeks follow-up (T2).
• Principal component analysis and Pearson’s correlation analysis to compare two cohorts.

## Results

### Study design and cohort-specific information

In the present work, two ethnically independent cohorts of COVID-19 patients were evaluated for their autoimmune response (total IgG-response) against 1,318 naturally folded human proteins (antigens). The Disc cohort was recruited at the ICU of HMC in Doha, Qatar, and included 49 COVID-19 cases and 48 healthy controls. A second cohort was recruited from the ICU of NYP Hospital, USA, which included 48 COVID-19 cases and 28 control patients and served as a Rep study. In addition, patients who were admitted to the NYP ICU and had infectious diseases other than COVID-19, such as bacterial sepsis ARDS or H1N1 pneumonia, were included as controls for the Rep cohort. Our studied cohorts predominantly included male subjects and only 13% were female subjects. Because of the unique composition of the cohorts, we were able to specifically look for COVID-19-related autoantibody signals compared with healthy-baseline- and general infection-baseline-titers. A combined analysis (discovery and replication) allowed stringent COVID-19-specific autoimmune responses to be monitored. Table 1 summarizes the demographic and status-specific information of the study cohorts.

**TABLE 1:** Summary metadata of COVID-19 cases and control cohorts.

Study specification	Condition	Discovery cohort (disc)	Replication cohort (rep)
Cohort size	Control	48	28
	COVID-19	49	48
	COVID-19 follow-up	15	
Gender (male and female)	Control	46 M + 2 F	19 M + 9 F
	COVID-19	48 M + 1 F	40 M + 8 F
	COVID-19 follow-up	15 M	
Age (IQR)	Control	38 (9)	66 (26)
	COVID-19	47 (20)	57 (22)
	COVID-19 follow-up	49 (11)	
BMI (mean (IQR))	Control	25.3 (9.2)	25.9 (9.4)
	COVID-19	29.9 (6.2)	28.9 (7.3)
	COVID-19 follow-up	28.7 (6.4)	
Sampling time (median days (IQR))	COVID-19	5 (3) days after ICU admission	6 (6) days after ICU admission
	COVID-19 follow-up	Six weeks after recovery	
Status controls		Healthy	ICU bacterial ARDS & pneumonia and ICU H1N1 ARDS & pneumonia
Status COVID-19		Severe	Severe, ARDS, pneumonia
Hospital		ICU, HMC, Qatar	ICU, New York, USA
Ethnicity (%)		South Asia (69), Middle East, North Africa (MENA) (25), and others (6)	White (31), Asian (10), African (9), and other/unspecified (50)
Matrix, tubes, and virus inactivation		Serum, non-EDTA-coated, viral inactivation using 10% Triton X-100	Plasma, EDTA-coated, viral inactivation using 10% Triton X-100

### General autoantibody response in healthy and COVID-19 patients

To discover functional IgG-related autoantibodies that could influence COVID-19 predictions and/or outcomes, we used the KREX high-throughput autoantibody assay technology that includes a variety of known human autoantigens such as cancer, kinase, interleukins, cytokine, ribonuclear transcription, and signaling proteins (Blackburn et al., 2020). Total IgG autoantibody responses were quantified for 1,600 proteins in the Discovery Cohort and a subset of 1,318 proteins in the Replication Cohort. However, to increase stringency and reduce complexity, only the 1,318 overlapping proteins were subsequently used in the analysis pipeline. The majority of antigens on the array are found in the cytoplasm, nucleus, or cell membrane, but there are also proteins from the mitochondria, endoplasmic reticulum, and cytoskeleton.

**FIGURE 1:**
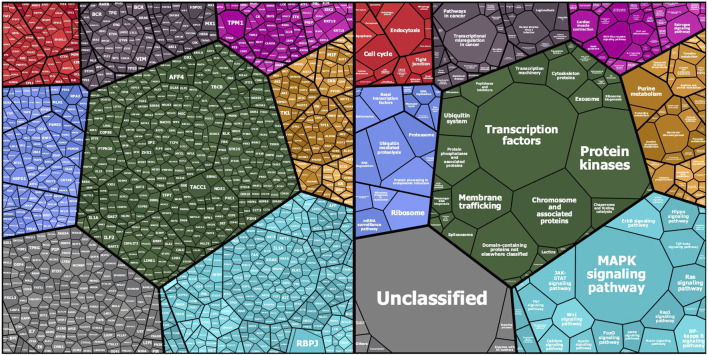
Mapping of KREX array proteins to KEGG categories (KEGG pathway and KEGG BRITE): Protein symbols and median fluorescent antibody signals (treemap cell size) are represented according their KEGG category assignment (www.kegg.jp; accessed on 14.Nov. 2021). The other main categories are defined as cellular processes (top left—red), human diseases (top middle—grayish purple), organismal systems (to right—magenta), genetic information processing (left—blue), BRITE protein families (center—dark green), metabolism (right—orange), and environmental information processing (bottom right—cyan). Unmapped proteins are considered “not included in pathway or BRITE” (bottom left—gray).

The KREX assay reports RFU values for autoantigen-specific autoantibody binding, with linearity over six orders of magnitude and a detection limit in the pg/ml range. These measured RFU values correlate directly with the antigen-specific IgG autoantibody titers. Ligand-binding theory shows that the measured signal on the array is linearly proportional to autoantibody concentration. Thus, a higher RFU value for a specific autoantibody–autoantigen interaction indicates a higher autoantibody titer, while a higher antibody titer, in turn, might imply a higher autoantigen concentration, which could be determined by gene or protein expression analysis in the tissue where it is expressed. In the first overview, the general intensity distributions were calculated based on the mean autoantibody–antigen titers across all samples and were further examined using KEGG-BRITE-based Voronoi treemaps using the replication cohort as an example (Figure 1). Approximately 1,150 of the 1,318 proteins were assigned to the annotation, with the relative size of each cell on the Voronoi treemaps reflecting the observed autoantibody response against that protein (Figure 1 left). Nearly all proteins showed a total IgG AB signal in the cases and the corresponding controls. The latter represents the natural autoimmunity or the healthy repertoire of autoantibodies. In Figure 1 (right), the corresponding pathways are summarized in different colors, with most proteins belonging to the MAPK pathway (light blue), followed by transcription factors (green), chromosomal proteins (green), ribosomes (all blue), and metabolic proteins (yellow). A few proteins belong to the cell cycle (red), chemokines (cyan), or cancer (black). The 10 highest autoantibody titers were found against RBPJ, TPM1, TACC1, KRT19, PTPN20, TBCB, KRT15, AFF4, HSPD1, and CBFA2T3, where many of these are structure-related proteins. The 10 proteins with the lowest titers were AIF1, IL18, NCK1, COMMD3, NEK11, TGFBR2, SLA, PKM, MAPK6, and MLKL, many of which are cytoplasmic proteins involved in phosphorylation.

### Disc cohort revealed significantly higher levels of SPANXN4 and ATF4 autoantibody response

**FIGURE 2:**
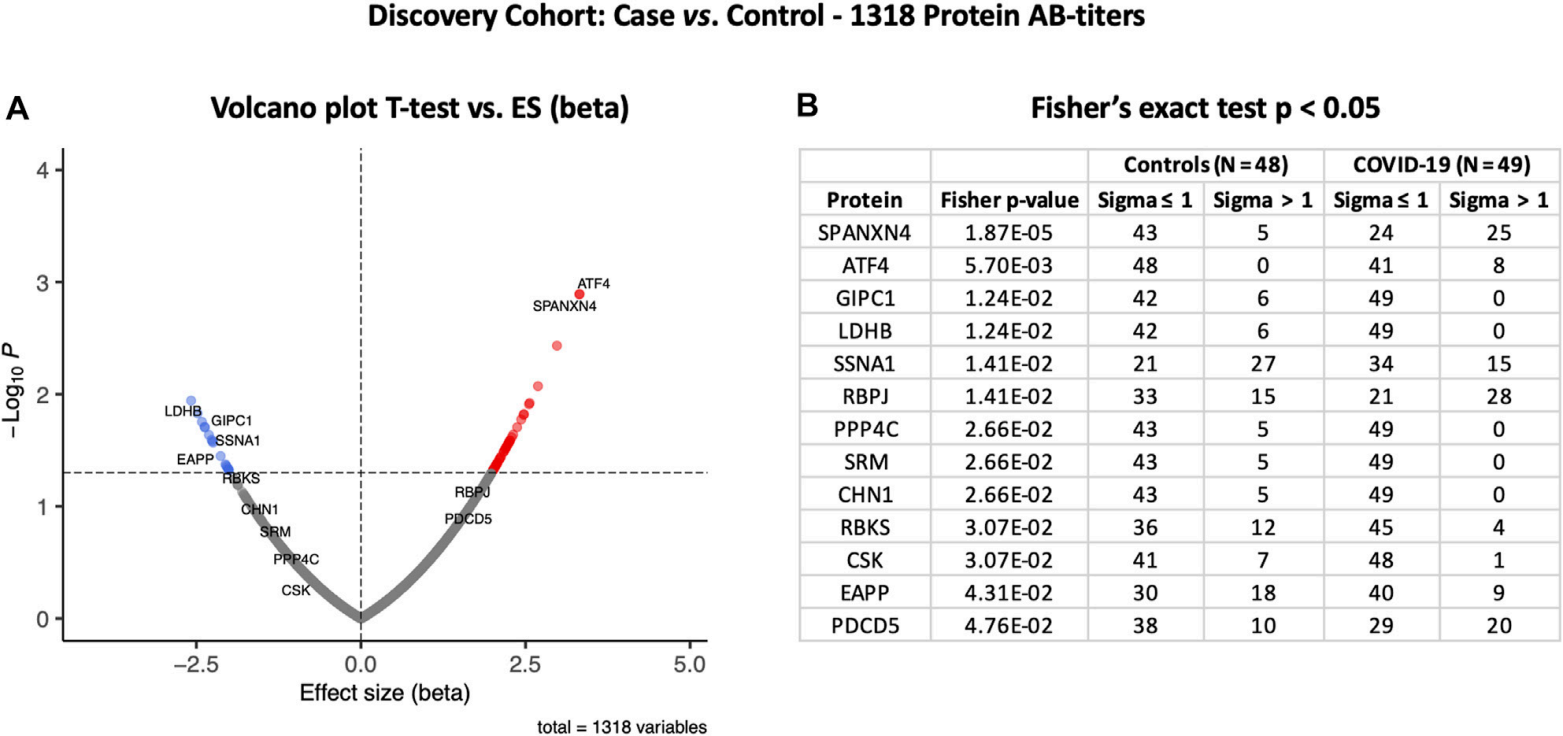
Differential protein autoantibody response analysis of the COVID-19 Discovery cohort performed using t-test **(A)** and Fisher’s exact test **(B)**. **(A)** Volcano graph of 1,318 proteins comparing the COVID-19 case (*n* = 49) vs. healthy controls (*n* = 48). Red dots represent proteins with an elevated autoantibody response, while blue dots represent proteins with a lower autoantibody response in COVID-19 patients. Proteins with Fisher’s test *p*-value ≤ 0.05 are labeled in the volcano graph. **(B)** Table on Fisher’s exact statistics comparing subjects (numbers) of COVID-19 (*n* = 49) and the control (*n* = 48) groups for only 13 proteins that showed significantly altered (*p*-value ≤ 0.05) autoantibody responses at sigma > 1.

To examine the effects of SARS-CoV-2 infection on the autoantibody response, we first performed a differential expression analysis in the Disc cohort between COVID-19 cases and healthy controls using the t-test (Supplementary Table S1). Autoantibody responses of 57 proteins were altered significantly (t-test *p*-value ≤ 0.05). Autoantibody responses in COVID-19 patients increased for 40 proteins and decreased for seventeen (Figure 2A). The most elevated autoantibody responses in COVID-19 patients were against ATF4 (effect size (beta) = 3.32 SD; t-test *p*-value ≤ 0.001) and the sperm protein associated with the nucleus on the X chromosome N4 (SPANXN4) (effect size (beta) = 3.32 SD; T-test *p*-value ≤ 0.001). The latter is also known as spermiogenesis-related protein and belongs to the family of cancer-/testis-associated proteins (CTAs) (Kouprina et al., 2004).

We then analyzed binarized autoimmune response, assuming all samples with an autoimmune response exceeding one s.d. as positive and all others as negative. Using Fisher’s exact test, we found that 25 COVID-19 patients had higher RFU values for SPANXN4 compared to only five in controls (Fisher’s test *p*-value ≤ 0.0001) (Figure 2B). Autoantibodies against ATF4, recombining signal-binding protein J (RBPJ), and programmed cell death 5 (PDCD5) were also significantly elevated (Fisher’s test *p*-value ≤ 0.05) in COVID-19 patients. Only the binarized SPANXN4 association reached the most stringent Bonferroni significance level, *p* < 0.05/number of proteins = 1,318. In the control group, EAPP, SSNA1, and LDHB proteins showed higher autoantibody responses than the cases.

### Autoantibody response in the disc follow-up cohort confirmed sustained high levels against SPANXN4 and other proteins in COVID-19 patients

**FIGURE 3:**
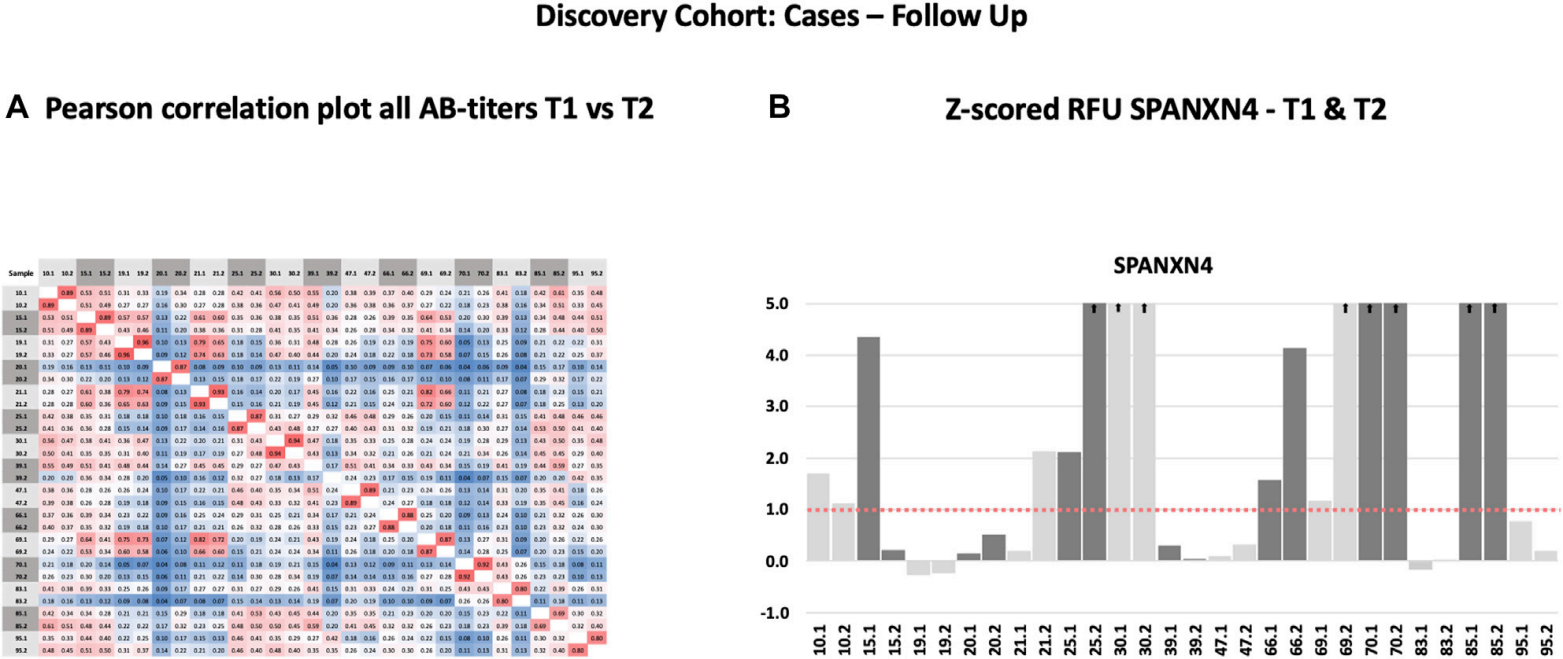
Autoantibody responses in the Discovery cohort (T1 = sampling during ICU admission) and follow-up patients (T2 = sampling after recovery). **(A)** Spearman’s rank correlation analysis of 15 Discovery cohort samples collected at two different time points shows strong correlation (r ≥ 0.69) of autoantibody responses for the proteins. **(B)** The histogram of Discovery cohort samples shows that the z-score RFU of SPANXN4 protein remains elevated in many patients even after COVID-19 recovery.

Following the initial blood sample collection at the time of ICU admission, follow-up samples were collected from 15 patients 6 weeks after recovery from COVID-19. For several proteins, a strong correlation (Pearson’s *r* ^2^ ≥ 0.69) was observed between the autoantibody responses at the two sampling time points (Figure 3A). Autoantibody responses against several proteins, including SPANXN4, STK25, TRAF3IP1, AMOTL2, PSMD4, and PPP1R2P9, remained highly elevated (*p* ≤ 0.05) at 6 weeks post-recovery follow-up. Particularly, autoantibody responses against SPANXN4 (Figure 3B) stayed elevated at both initial (T1) and follow-up (T2) time points. These observations reveal that SPANXN4 autoantibody responses remain elevated for extended periods, suggesting a potential association with chronic health issues.

### Relative autoantibody response in the Rep Cohort confirmed the trend in the Disc cohort

**FIGURE 4:**
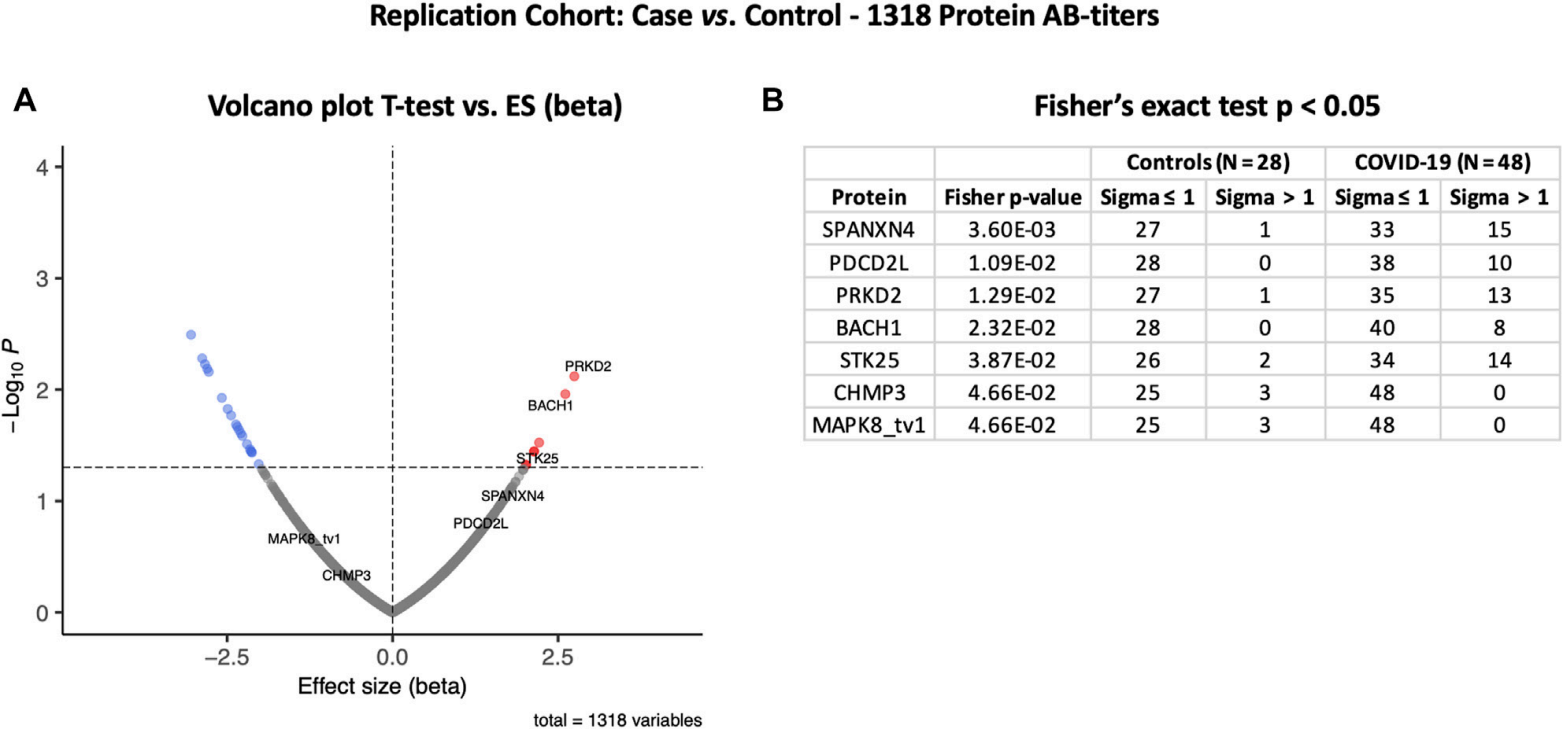
Differential protein autoantibody response analysis of the COVID-19 Replication cohort performed using t-test **(A)** and Fisher’s exact test **(B)**. **(A)** Volcano graph of 1,318 proteins comparing COVID-19 cases (*n* = 48) vs. non-COVID-19 ICU controls (*n* = 28). Red dots represent proteins with a high autoantibody response, while blue dots represent proteins with a low autoantibody response in COVID-19-positive patients. Only proteins with Fisher’s test *p*-value ≤ 0.05 are labeled in the volcano graph. **(B)** Table on Fisher’s exact statistics comparing subjects (numbers) of COVID-19 (*n* = 49) and the control (*n* = 48) groups for only 13 proteins that showed significantly altered (*p*-value ≤ 0.05) autoantibody responses at sigma > 1.

The autoantibody response for the Rep cohort (*n* = 48) was compared with that of the non-COVID-19 ICU control patients (*N* = 28) (Figure 4A). Autoantibody responses of 26 proteins altered significantly (t-test *p*-value ≤ 0.05) in the Rep cohort. Based on t-test analysis, the most elevated autoantibody response in the Rep COVID-19 cohort was found for PRKD2 and BACH1 proteins, which are known for their roles in male reproductive tract development (PRKD2) (Nie and Arend, 2014) and spermatogenesis (BACH1) (Yu et al., 2013). Autoantibody response for SPANXN4 was also higher [effect size (beta) = 1.61] in the Rep COVID-19 patients, albeit the *p*-value was slightly higher than 0.05.

However, Fisher’s exact test indicated that autoantibody response to SPANXN4 remained the highest (Fisher’s test *p*-value = 0.0036) (Figure 4B). At sigma 1, 15 COVID-19 patients had higher RFU values for SPANXN4 than only one in the controls. SPANXN4 can, therefore, be considered fully replicated under the highest standards of a discovery-replication design. PDCD2L, PRKD2, and STK25 also showed higher autoantibody responses in COVID-19 patients (Fisher’s test *p*-value ≤ 0.05).

### Analysis of combined cohorts supported that SPANXN4 and STK25 autoantibodies were significantly elevated in COVID-19 patients, independent of sampling matrix or patient ethnicity

**FIGURE 5:**
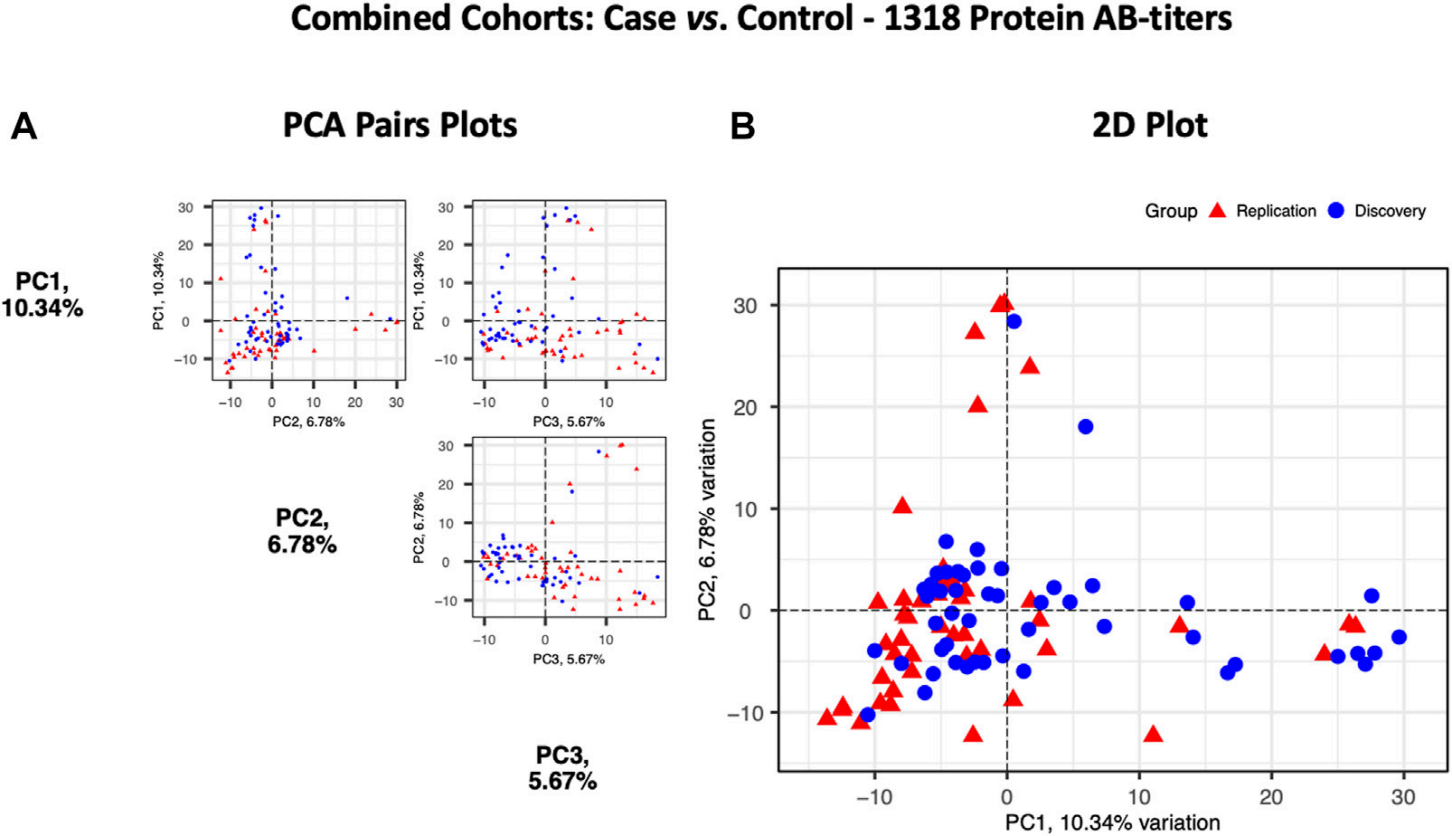
Principal components analysis of the Discovery (*n* = 49, blue circles) and the Replication cohorts (*n* = 48, red triangles). Each point represents a sample. **(A)** PCA pair plot comparing PC1 to PC3. The proportion of variance explained in our cohorts by each PC is shown in parentheses on the axis labels. **(B)** PCA 2D plot with PC1 and PC2, which together describe 17.12% diversity between the cohorts.

Principal components analysis (PCA) of protein RFU data from the two cohorts demonstrated a strong overlap between COVID-19 samples, and the two cohorts did not separate into discrete clusters (Figure 5). Pearson’s correlation analysis revealed that the autoantibody responses of the two cohorts had a high correlation (*r* ^2^ = 0.73).

**FIGURE 6:**
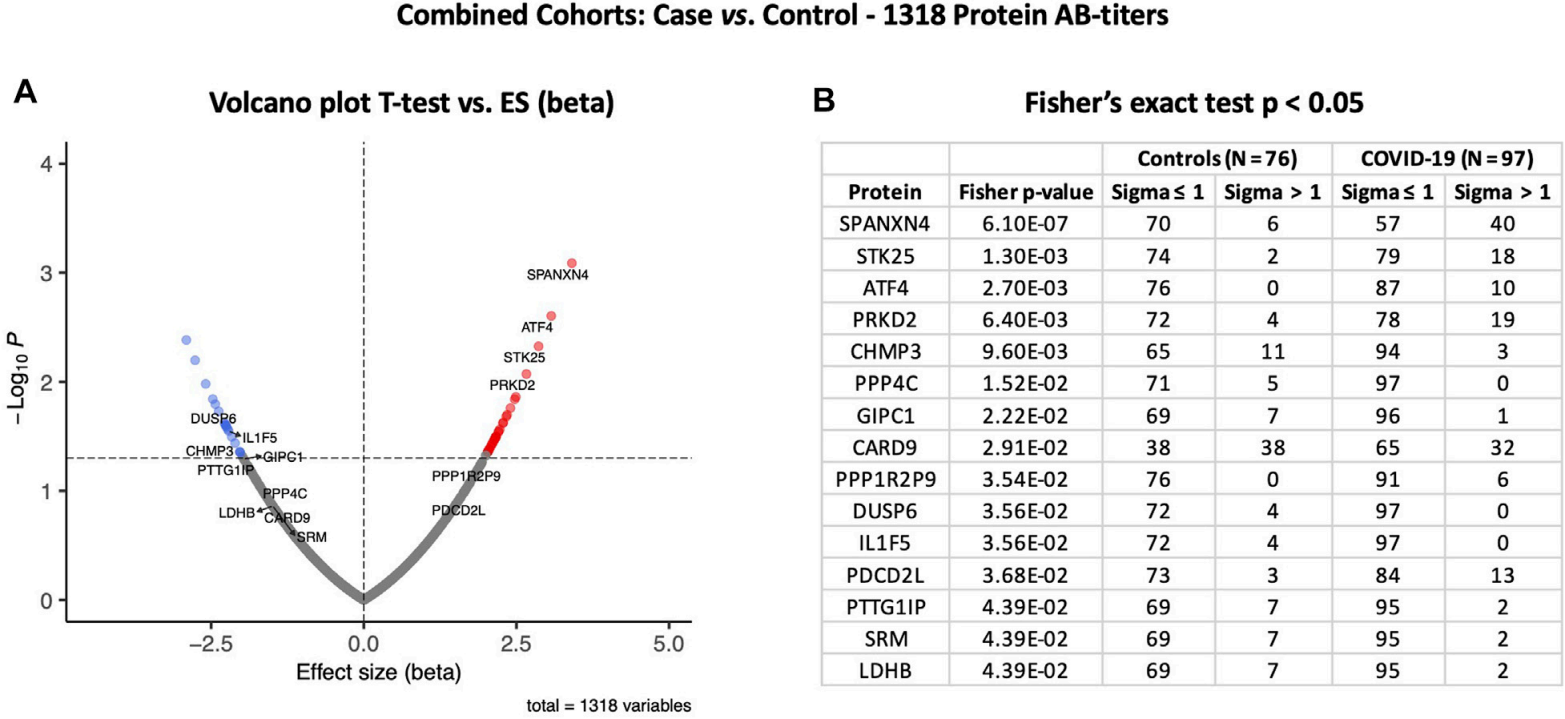
Differential protein autoantibody response analysis of combined cohorts performed using t-test **(A)** and Fisher’s exact test **(B)**. **(A)** Volcano graph of 1,318 AB-protein titers comparing COVID-19 cases (*n* = 97) vs. controls (*n* = 76). Red dots represent proteins with a high autoantibody response, while blue dots represent proteins with a low autoantibody response in COVID-19-positive patients. Only proteins with Fisher’s test *p*-value ≤ 0.05 are labeled in the volcano graph. **(B)** Table on Fisher’s exact statistics comparing subjects (numbers) of COVID-19 (*n* = 49) and the control (*n* = 48) groups for only 13 proteins that showed significantly altered (*p*-value ≤ 0.05) autoantibody responses at sigma > 1.

At the third stage, we combined data from both the Disc and Rep cohorts (*n* = 97) and compared them with those of combined controls (*n* = 76). Case vs. control analysis revealed that autoantibody responses against 56 proteins were significantly altered: 35 autoantibodies with increased and 21 autoantibodies with decreased responses (t-test *p* ≤ 0.05) (Figure 6A). SPANXN4, ATF4, STK25, and PRKD2 were the proteins with the highest effect size (beta). In total, 40 patients had SPANXN4 RFU values higher than 1 sigma value (Fisher’s exact test *p*-value ≤ 0.0001) in the combined COVID-19 cohorts compared with six patients only in controls (Figure 6B).

**FIGURE 7:**
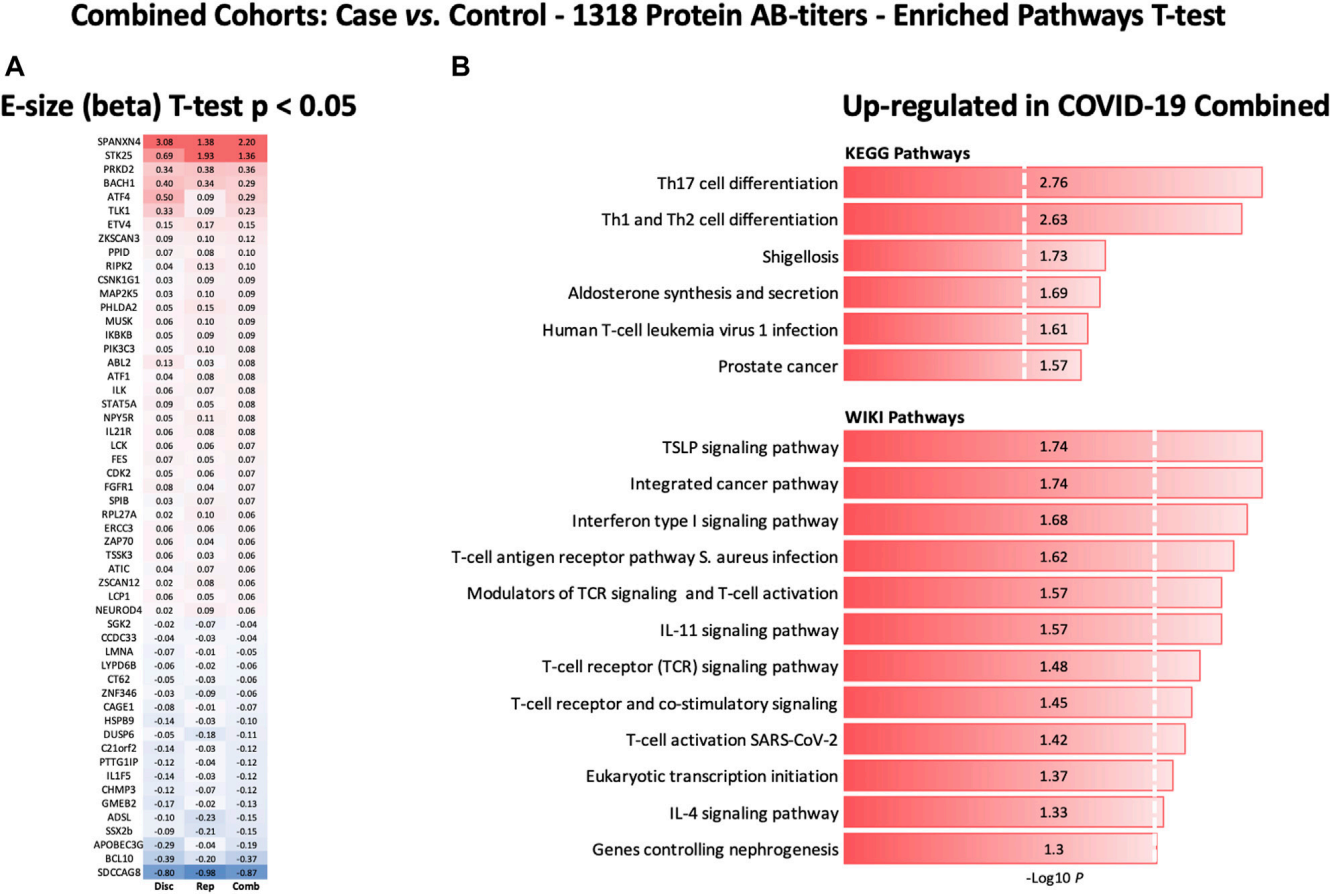
Heatmap of autoantibody response pattern among different cohorts **(A)** and bar plots for pathway analysis. **(A)** Heatmap of relative estimates (case vs. control) of autoantibody responses to 56 proteins in the Discovery, Replication, and Combined cohorts. Only proteins with significantly altered autoantibody responses were selected. Red color indicates higher autoantibody responses, and blue color indicates lower autoantibody responses against the proteins. **(B)** KEGG and WIKIPathways analyses presented as bar-plot shows over-activated pathways in COVID-19 patients. Only pathways with t-test *p*-value ≤ 0.05 are presented in the bar plot.

Furthermore, the autoantibody responses expressed as RFU z-score for 56 proteins that differed significantly between the study groups are shown in Figure 7A. The heatmap shows that most of the proteins display similar patterns of autoantibody ratios across the study cohorts. These analyses demonstrate that our autoantibody response data are highly reproducible, despite differences in population ethnicity, different laboratories, and sampling materials (serum vs. plasma in Disc vs. Rep cohorts, respectively).

**FIGURE 8:**
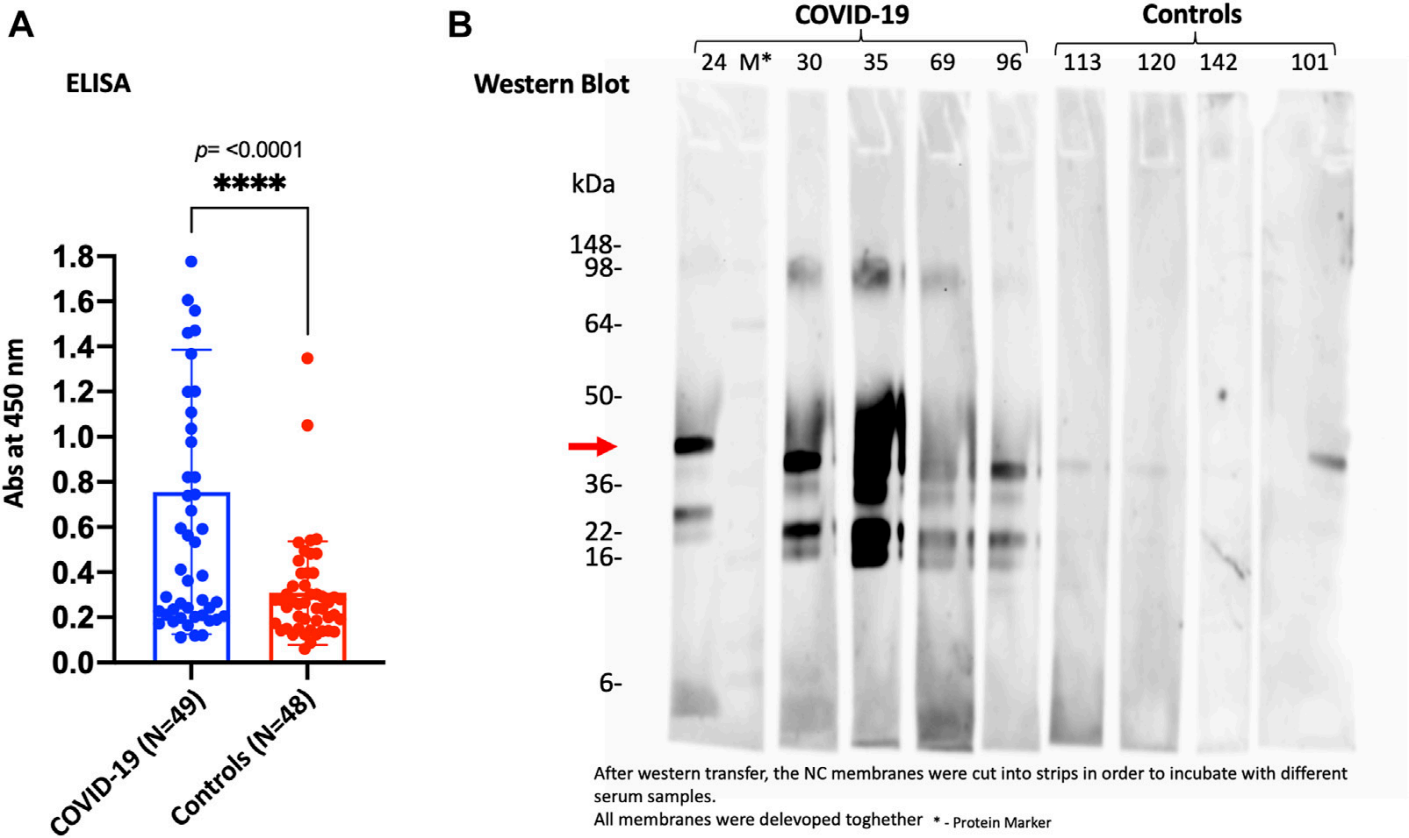
ELISA **(A)** and Western blot **(B)** performed for SPANXN4 protein for Disc cohort samples **(A)** to confirm Sengenics KREX RFU data. **(A)** COVID-19 vs. control sample SPANXN4 absorbances were compared using unpaired t-test. **(B)** Western blot of the randomly selected COVID-19 and control samples shows increased autoantibodies against SPANXN4 where all tested COVID-19 serum samples detected the SPANXN4 protein with a high signal compared to control samples.

On the other hand, to validate and confirm the KREX data showing significantly increased levels of SPANXN4, we performed an indirect ELISA to determine the level of SPANXN4 autoantibodies in serum samples from Disc COVID-19 cases. ELISA plates were coated with SPANXN4 proteins and probed with 1/400 diluted serum samples obtained from severe COVID-19 cases and controls. Using ELISA, we found that SPANXN4 autoantibodies were significantly higher in serum samples obtained from COVID-19 cases than from controls (*p* ≤ 0.0001) (Figure 8A). We further confirmed the increased autoantibodies against SPANXN4 using Western blot analysis, where all tested COVID-19 serum samples detected the SPANXN4 protein with a high signal compared to control samples (Figure 8B). Overall, these validation assays agree with the KREX data showing that the most elevated autoantibody response in COVID-19 patients was against SPANXN4.

### Protein pathway analysis uncovered upregulated immune pathways in COVID-19 patients

KEGG and WIKIPathways analyses were performed to identify the functional contribution of autoantibody targeted proteins in cellular processes and immune-inflammatory systems. Pathways associated with T helper cell (Th1, Th2, and Th17) differentiation, bacterial/viral infections, stress hormone release, and prostate cancer were upregulated in COVID-19 patients (Figure 7B). WIKIPathways was also activated for host immunity and interferon signaling, including T-cell activation for SARS-CoV-2 and *Staphylococcus aureus* infections (Figure 7B).

### SPANXN4 and STK25 share sequence identity with SPANX and STK family proteins but showed unique AB-titers in COVID-19 patients

**FIGURE 9:**
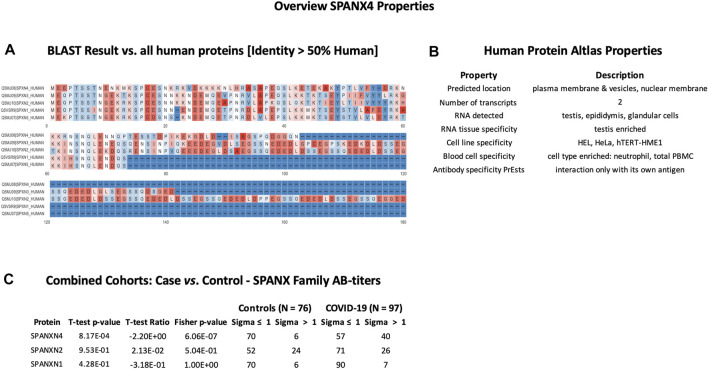
SPANXN4 protein sequence identity and antigen specificity analysis: **(A)** sequence alignment of proteins showing ≥ 50% identity with SPANXN4. **(B)** Summary of properties of SPANXN4 protein from the Human Protein Atlas. **(C)** Autoantibody responses to SPANXN family proteins with high sequence identity to SPANXN4 that were part of the KREX Immunome panel.

**FIGURE 10:**
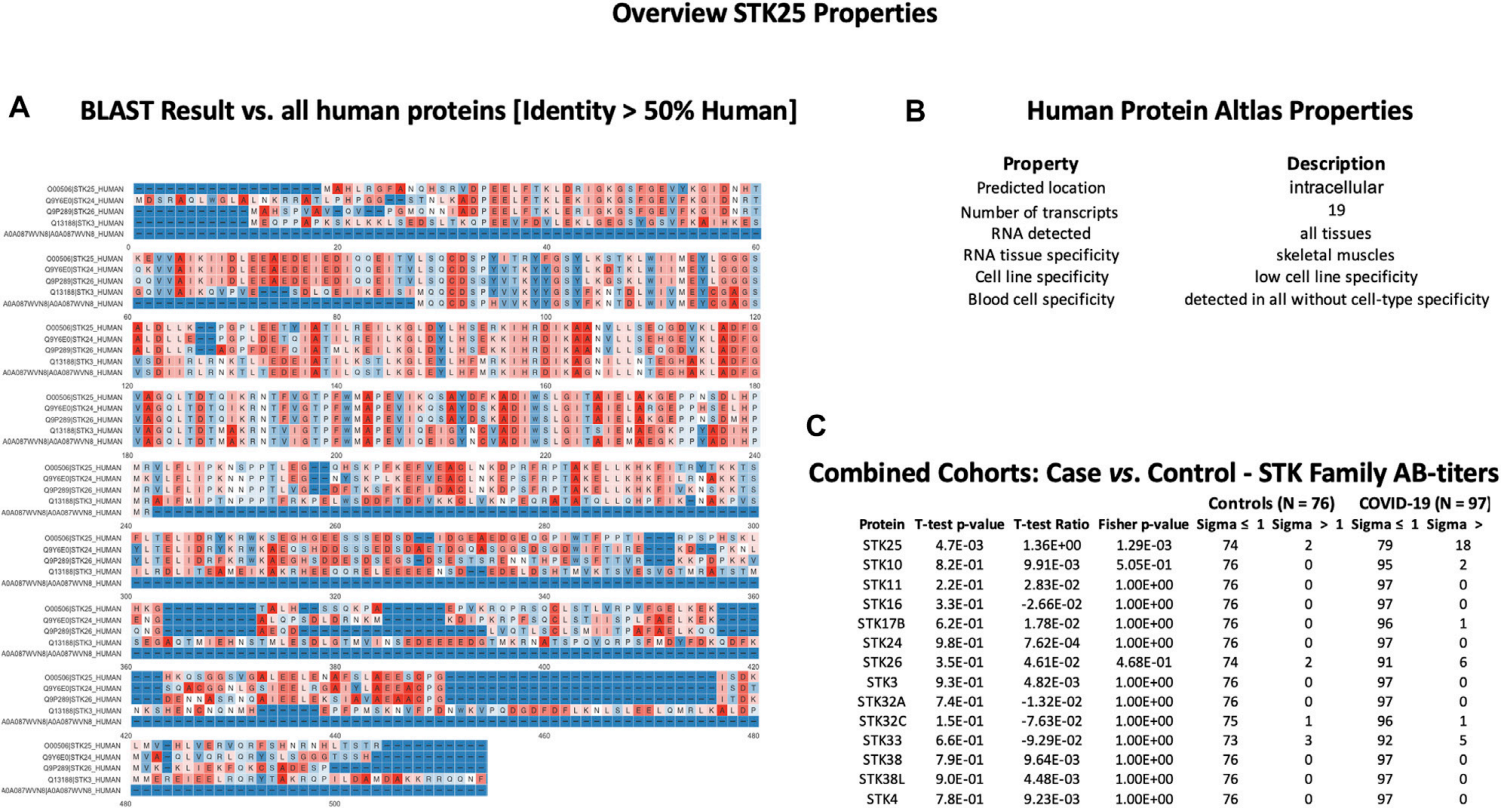
STK25 protein sequence identity and antigen specificity analysis: **(A)** sequence alignment of proteins showing ≥ 50% identity with STK25. **(B)** Summary of properties of STK25 protein from the Human Protein Atlas. **(C)** Autoantibody responses to STK family proteins with high sequence identity to SKT25 that were part of the KREX Immunome panel.

In order to check cross-reactivities, sequence homology and antigen-specificity analysis were performed for SPANXN4 (Figure 9A) and STK25 (Figure 10A) against human and viral protein databases. Only a few proteins appeared to have more than 50% sequence identity with our target proteins ((SPANXN4 with SPANXN1, 2, 3, and 5) and (STK25 with STK 3, 4, 24, and 26)) (Figures 9, 10). Proteins characteristics are summarized along with their functions and locations (Figures 9B, 10B). However, many of these homologous proteins were also part of our KREX Immunome panel. However, they showed no significant changes (Figures 9C, 10C), meaning the observed RFUs are highly specific against the targeted proteins.

## Discussion

In the current COVID-19 pandemic, there has been increasing global interest in understanding the underlying immunology of COVID-19 and revealing new health issues arising from COVID-19 complications. Several papers have described the existence and cross-reactivity of SARS-CoV-2-specific T-cell responses (Grifoni et al., 2020; Peng et al., 2020; Reynolds et al., 2020; Sekine et al., 2020) and correlations with the male reproductive system and infertility (Sun, 2020; Delle Fave et al., 2021). The present study identified and validated several autoantibody responses by screening two independent cohorts of COVID-19 patients with the KREX Immunome protein array. The proteins identified with higher autoantibody responses serve important physiological functions and are strongly associated with various immunological and pathological parameters associated with the COVID-19 disease.

The KREX Immunome array contains proteins involved in physiological processes such as MAPK signaling, metabolism, transcription, cell cycle, immunity, and cancer-related pathways. A few of the proteins with the highest mean autoantibody response in COVID-19 patients were RBPJ, TPM1, TACC1, KRT19, and PTPN20. These proteins perform various physiological functions in the human body, many of which are structural proteins involved in tissue damage and repair mechanisms (Zhang et al., 2019). A high autoantibody response to these proteins suggests that they are overproduced during a pathological condition, such as cancer or a cardiovascular complication (Lv et al., 2015; Saha et al., 2017). For example, the Notch signaling protein RBPJ has been associated with COVID-19 pathophysiology and cardiovascular complication (Breikaa and Lilly, 2021). Similarly, keratin family proteins (KRT19 and KRT15) are responsible for epithelial cell structural integrity and are linked to COVID-19 pathogenesis and disease severity (Gisby et al., 2021). Furthermore, many of these proteins are also involved in male reproductive system physiology and fertility, yet there has been no previous report on COVID-19 patients.

Our Disc cohort reported higher autoantibodies against SPANXN4, ATF4, RBPJ, and PDCD5 proteins compared to the controls. Comparison between the COVID-19 baseline (T1) and follow-up (T2) samples indicated that the number of SPANXN4 autoantibodies remained elevated at the post-recovery stage. Prolonged autoantibody responses may highlight COVID-19 post-acute sequelae by stimulating the humoral immune response in a way that leads to long-term autoantibody production (Proal and VanElzakker, 2021). The diverse variety of proteins linked to a prolonged autoantibody response suggests that SARS-CoV-2 may stimulate autoantibody formation by molecular mimicry (Liu et al., 2021), targeting cardiolipin, cardiolipin-binding proteins, platelet factor 4, prothrombin, and coagulation factors, therefore suggesting their role in coagulopathies, chronic comorbidities, and post-infection recovery (Leng et al., 2020; Mishra et al., 2020; Root-Bernstein, 2021), We hypothesize that elevated autoimmune antibodies against SPANXN4, STK25, TRAF3IP1, AMOTL2, PSMD4, and PPP1R2P9 might suggest a similar role. However, Dotan et al. (2021) investigated *in silico* sequence homology of all human proteins with the virus and could not find evidence that any of the proteins mentioned here are part of such a mimicry process. Vice versa, we cannot exclude that the titers might be elevated before exposure to SARS-CoV-2 due to pre-existing diseases such as cancer or prolonged inflammation.

In contrast, the Rep cohort had higher autoantibody responses to SPANXN4, PDCD2L, PRKD2, and STK25 proteins than the controls. Except for SPANXN4, all other proteins with a high autoantibody response were not significantly elevated between the two cohorts but often showed similar trends. These differences could be attributed to the fact that the control group in the Disc cohort comprised healthy volunteers. In contrast, the Rep study’s control group comprised ICU patients suffering from bacterial or viral ARDS or pneumonia. To that purpose, comparing COVID-19 patients’ data with those of two categorically different controls offers a new horizon for evaluating and confirming our analytes’ cross-infection and disease states.

When all COVID-19 patients (*N* = 97) from both cohorts were merged and compared to all controls (*N* = 76) from both cohorts, the most significant autoantibody responses were observed against SPANXN4, ATF4, STK25, and PRKD2. ATF4 is a transcription factor that activates the expression of IL-8, CHOP, MCP1, HERP1, and IGFBP-1 genes in response to endoplasmic reticulum stress (Gargalovic et al., 2006; Marchand et al., 2006). ATF4 regulates metabolic and redox processes in the human body, and an increased ATF4 response has been observed in previous coronavirus pandemics (Köditz et al., 2007; Liao et al., 2013). ATF4 increases the expression of genes involved in the adaptive response to stress in COVID-19 (Rosa-Fernandes et al., 2021). Fischer et al. (2004) suggested that ATF4 also plays a role in the differentiation of the vas deferens lamina propria layer that helps improve spermatozoa fertilization rate. STK25 and PRKD2 are important kinases with several physiological roles in our bodies. However, their role in male reproductive tract physiology is least discussed. A few studies highlight STK25 as an androgenic kinase (Hammes et al., 2005; Gill-Sharma, 2018) and the role of PRKD2 in male reproductive tract development (Dai et al., 2019).

SPANXN4 belongs to a protein family called “sperm protein associated with the nucleus in the X chromosome” (SPANX) that is essential for the motility and fertilization capacity of male-ejaculated spermatozoa (Urizar-Arenaza et al., 2020). SPANXs have been reported to be expressed throughout the process of spermatogenesis from gamete cell precursors to ejaculated spermatozoa, indicating their association with sperm development (Hansen et al., 2010). SPANXNs are also known as cancer-testis antigens (CTAs) because of their overexpression in tumor tissues, and due to their normal physiological role in the testis and spermatozoa of healthy males (Zendman et al., 2003). Because SPANXN4 is a male genital-related protein, our discovery-driven study had a limitation for including a small number of female subjects. Nevertheless, SPANXN4 autoantibodies had very high levels in independent multi-ethnic cohorts, so we can assume that analyzing the levels of SPANXN4 autoantibodies in only male subjects (predominant) should provide similar results.

Using proteomics analysis, Ghosh et al. (2022) recently discovered that COVID-19 convalescent men had de-regulated proteins and molecular pathways related to male reproductive function. Interestingly, one SPANX family protein, SPNXC, was upregulated in COVID-19 convalescent men (Ghosh et al., 2022). Here, we are reporting for the first time that autoantibodies against SPANX proteins are found elevated in severe COVID-19 and recovered patients. These elevated immune responses against SPANXN4 proteins indicate possible de-regulation of sperm functions. No previous COVID-19 study has mentioned SPANXN4 involvement in male infertility, and neither has the pathogenesis been explained. Therefore, the autoantibody response measured in the current study may suggest a novel diagnostic and treatment marker for male fertility. Previously, one hepatitis C virus study showed that SPANXN4 interacts with the virus, potentially increasing virus infectivity, albeit no reproductive performance was discussed (Ngo et al., 2013). Increased levels of autoantibodies against testis-related proteins suggest their role in affecting the male reproductive system and, thus, declining male fertility in COVID-19. Although several investigations have found that COVID-19 patients have altered seminal parameters and decreased reproductive hormone levels (Khalili et al., 2020), histological or functional abnormalities in the male genital system (Youssef and Abdelhak, 2020), damaged blood–testis barrier (Peirouvi et al., 2021), and impaired spermatogenesis (Li et al., 2020; Gharagozloo et al., 2022), the cause of this comorbidity has not yet been investigated and remains unknown. In addition to COVID-19 infection, vaccination with BNT162b2, which induced humoral and cell-mediated immune response against spike protein, may impact fertility and temporarily influence the semen concentration and motile count among semen (Agrati et al., 2022).

Despite the ethnic diversity of our cohorts, which included Middle Eastern, African, Caucasian, Asian, and South Asian populations, correlation analysis and hierarchical and PCA clustering demonstrated that both cohorts shared similarities in autoantibody responses. Therefore, a strong correlation (*r* ^2^ = 0.73) between these cohorts demonstrates that autoantibody response data of COVID-19 patients are highly reproducible among different ethnic populations. These findings are consistent with those of our prior COVID-19 proteomics study examining immune-inflammatory markers in five different demographic cohorts (Suhre et al., 2021).

Several previous COVID-19 studies have reported elevated immune-inflammatory responses, including cytokine storm in COVID-19 patients. Perhaps, we observed relatively elevated, albeit non-significant, autoantibody responses to immune cytokines like IL1A and IL1B proteins. KEGG and WIKIPathways analyses showed that autoantibody responses to immune proteins activated T-cell responses against infection and T helper cell differentiation. Li et al. (2021) observed that Th17 differentiation and cytokine response pathways play a crucial role in the pathogenesis of COVID-19 and autoimmune diseases. Pathway analysis suggests that many immune cell responses specific to SARS-CoV-2 or bacterial infections may precede chronic inflammatory disorders and respiratory failure (Ouyang et al., 2020). Furthermore, an abnormal T helper cell response and overactive interferon signaling promote the differentiation of B cells, which produce autoantibodies and cause autoimmune diseases (Zhang et al., 2020).

In conclusion, these findings reveal unique autoantibody responses against several proteins that play diverse, though important, functions in COVID-19 complications. These observations also highlight the humoral immune response’s importance and numerous other previously unknown immunological pathways in COVID-19 pathogenesis. In both cohorts, elevated levels of autoantibodies against the testicular tissue-specific protein SPANXN4 offer significant evidence of anticipating the protein’s role in COVID-19-associated male reproductive complications. These findings not only revalidate autoantibody responses against SPANXN4 in COVID-19 but also predict novel pathological associations that may contribute to COVID-19 post-recovery comorbidities. SPANXN family proteins, known as CTAs (Chiriva-Internati et al., 2008), play an essential role in spermatogenesis. However, their role in male fertility in COVID-19 patients needs to be validated further using mechanistic models.

## Data Availability

The original contributions presented in the study are included in the article/Supplementary Material; further inquiries can be directed to the corresponding authors.
